# BjMYB1, a transcription factor implicated in plant defence through activating *BjCHI1* chitinase expression by binding to a W-box-like element

**DOI:** 10.1093/jxb/erw240

**Published:** 2016-06-27

**Authors:** Ying Gao, Shuangwei Jia, Chunlian Wang, Fujun Wang, Fajun Wang, Kaijun Zhao

**Affiliations:** National Key Facility for Crop Gene Resources and Genetic Improvement (NFCRI), Institute of Crop Science, Chinese Academy of Agriculture Sciences (CAAS), Beijing 100081, China

**Keywords:** BjMYB1, *Botrytis cinerea*, fungus, MYB transcription factor, pathogen defence, W-box-like element.

## Abstract

We previously identified the W-box-like-4 (Wbl-4) element (GTAGTGACTCAT), one of six Wbl elements in the BjC-P promoter of the unusual chitinase gene *BjCHI1* from *Brassica juncea*, as the core element responsive to fungal infection. Here, we report the isolation and characterization of the cognate transcription factor interacting with the Wbl-4 element. Using Wbl-4 as a target, we performed yeast one-hybrid screening of a *B. juncea* cDNA library and isolated an R2R3-MYB transcription factor designated as BjMYB1. BjMYB1 was localized in the nucleus of plant cells. EMSA assays confirmed that BjMYB1 binds to the Wbl-4 element. Transiently expressed BjMYB1 up-regulated the activity of the BjC-P promoter through its binding to the Wbl-4 element in tobacco (*Nicotiana benthamiana*) leaves. In *B. juncea, BjMYB1* displayed a similar induced expression pattern as that of *BjCHI1* upon infection by the fungus *Botrytis cinerea*. Moreover, heterogeneous overexpression of *BjMYB1* significantly elevated the resistance of transgenic *Arabidopsis thaliana* to the fungus *B. cinerea*. These results suggest that BjMYB1 is potentially involved in host defence against fungal attack through activating the expression of *BjCHI1* by binding to the Wbl-4 element in the BjC-P promoter. This finding demonstrates a novel DNA target of plant MYB transcription factors.

## Highlight

BjMYB1, an R2R3-MYB protein from *Brassica juncea,* binds to W-box-like elements rather than AC elements to mediate plant defence against fungus.

## Introduction

Plants are sessile organisms and therefore vulnerable to various pathogens in their habitat. Among the pathogens encountered, fungi pose a widespread threat to the conservation of plant species and food security of human beings (Fisher *et al.*, 2012). It has been estimated that fungal diseases of the five major food crops—rice (*Oryza sativa*), wheat (*Triticum aestivum*), corn (*Zea mays*), soybean (*Glycine max*), and potato (*Solanum tuberosum*)—cause a yield loss of 125 million tons yearly (Fisher *et al.*, 2012). For example, *Botrytis cinerea*, the second most important plant fungal pathogen, infects more than 200 plant species and causes massive economic losses in agriculture (Dean *et al.*, 2012; Lu *et al.*, 2013; Weiberg *et al.*, 2013).

Over the course of evolution, plants have evolved sophisticated mechanisms to defend against pathogen attack (Jones and Dangl, 2006; Lu *et al.*, 2013). In responding to pathogen infection, plant genes for defence are activated, and transcription factors (TFs) play important roles (Windram *et al.*, 2012; Liu *et al.*, 2013). The major TFs that activate plant defence genes against *B. cinerea* are the WRKY and AP2/ERF families (Berrocal-Lobo *et al.*, 2002; Chen and Chen, 2002; Dong *et al.*, 2003; Lai *et al.*, 2008; Hu *et al.*, 2012). A number of investigations have revealed that the highly conserved WRKY domains in most WRKY TFs specifically bind to the W-box elements characterized by the nucleotide motif (T/C)TGAC(C/T) (Eulgem *et al.*, 2000; Eulgem and Somssich, 2007; Rushton *et al.*, 2010; Chi *et al.*, 2013), although some recent reports have shown that WRKY proteins also display binding affinity to various W-box-like (Wbl) elements in which only a central GAC-core motif is required for binding (Ciolkowski *et al.*, 2008; Yamasaki *et al.*, 2012; Brand *et al.*, 2013).

MYB proteins comprise the largest family of TFs in plants and play regulatory roles in plant development, secondary metabolism, hormone signalling, disease resistance, and abiotic stress tolerance (Katiyar *et al.*, 2012). MYB TFs have been classified into four subfamilies (R1-MYB, R2R3-MYB, 3R-MYB, and 4R-MYB) based on the MYB domains they contain (Dubos *et al.*, 2010). An R2R3-MYB protein contains two MYB domains and the majority of R2R3-MYB proteins regulate plant-specific functions including immunity against microbial pathogens (Stracke *et al.*, 2001; Dubos *et al.*, 2010). For example, overexpression of the R2R3-MYB gene *HbMyb1* from *Hevea brasiliensis* enhances resistance to *B. cinerea* in transgenic tobacco (*Nicotiana tabacum* cultivar Samsun NN; Peng *et al.*, 2011); the wheat R2R3-MYB gene *TaPIMP1* regulates plant resistance to the biotrophic bacterial pathogen *Ralstonia solanacearum* in tobacco (*Nicotiana tabacum* L.) and to the hemibiotrophic fungal pathogen *Bipolaris sorokiniana* in wheat (Liu *et al.*, 2011; Zhang *et al.*, 2012); and the R2R3-MYB gene *OsJaMyb* in rice (*O. sativa* spp. *japonica*) is responsive to infection by the blast fungus *Magnaporthe oryzae* (Lee *et al.*, 2001). Moreover, *AtMYB30*, the most extensively characterized R2R3-MYB gene in *Arabidopsis thaliana*, is involved in the regulation of plant immunity to microbial pathogens (Raffaele and Rivas, 2013). Some other R2R3-MYB genes from *A. thaliana,* such as *BOTRYTIS-SUSCEPTIBLE1 BOS1/AtMYB108* (Mengiste *et al.*, 2003), *AtMYB72* (Segarra *et al.*, 2009), and *AtMYB60* and *AtMYB96* (Seo *et al.*, 2009; Seo and Park, 2010), are also associated with the regulation of plant resistance to pathogens.

To unlock the prominent roles played by R2R3-MYB TFs in plant defence against pathogen attack at the molecular level, it is necessary to investigate the details of the interaction between R2R3-MYB TFs and their target genes (Prouse and Campbell, 2013). Investigations have shown that the MYB-core element (C/T)NGTT(G/A) and the AC elements ACC(A/T)A(A/C)(T/C) and ACC(A/T)(A/C/T)(A/C/T) are *cis*-regulatory elements of R2R3-MYB proteins for transcriptional activation of target genes in yeast and *in planta* (Romero *et al.*, 1998; Prouse and Campbell, 2012; Kelemen *et al.*, 2015). Grotewold and colleagues first reported the ACC(A/T)ACC(A/C/T) target site of the maize P protein, an R2R3-MYB protein involved in flavonoid biosynthesis (Grotewold *et al.*, 1994). Likewise, the flavonoid biosynthesis-associated proteins AtMYB11, AtMYB12, and AtMYB111 show similar target gene specificity as the maize P protein (Prouse and Campbell, 2012). The *Zea mays* MYB31 protein has been shown to bind to the sequence ACC(T/A)ACC within promoters of the genes *ZmCOMT* and *ZmF5H* (Fornalé *et al.*, 2010). Similarly, pine (*Pinus taeda*) MYB1 (Patzlaff *et al.*, 2003*b*) and MYB4 (Patzlaff *et al.*, 2003*a*), eucalyptus (*Eucalyptus grandis*) MYB2 (Goicoechea *et al.*, 2005), and AtMYB61 (Prouse and Campbell, 2013) also bind to AC elements in promoters of the lignin biosynthetic genes. However, to the best of our knowledge, no one has shown that an MYB protein can bind to a W-box or a Wbl element to regulate plant defence against fungal infection.

The W-box and/or Wbl elements are a major class of *cis*-acting elements in promoters of many plant genes responsive to pathogen induction (Rushton *et al.*, 1996; Rushton and Somssich, 1998; Rushton *et al.*, 2002; Yamamoto *et al.*, 2004; Gao *et al.*, 2014). The typical nucleotide motif (T/C)TGAC(C/T) of W-box elements is usually bound by the WRKY TFs (Eulgem *et al.*, 2000; Eulgem and Somssich, 2007; Rushton *et al.*, 2010; Chi *et al.*, 2013). For example, rice OsWRKY53 mediates the chitin elicitor-responsiveness by interacting with three tandem W-box elements (Chujo *et al.*, 2009). In our previous study, we identified six Wbl sequences in the chitinase gene *BjCHI1* promoter (BjC-P) and designated them as Wbl-1 through Wbl-6 (Wu *et al.*, 2009). Our further study showed that Wbl-4 (GTAGTGACTCAT) in the promoter BjC-P is the core element responsive to fungal infection (Gao *et al.*, 2014). However, the cognate TF interacting with the Wbl-4 element remains unknown. Here, we report the isolation and characterization of an R2R3-MYB TF (BjMYB1) that interacts with the Wbl-4 element to regulate plant defence against the fungus *B. cinerea*. To our knowledge, this is the first report that an MYB protein can bind to a Wbl element, rather than AC elements, to mediate plant pathogen defence.

## Materials and methods

### Plant materials and growth conditions

Plants of *A. thaliana* L. (ecotype Col-0), *Nicotiana benthamiana*, and *B. juncea* were grown in a growth chamber at 22°C (light)/19°C (dark) under a 16h light/8h dark cycle. The *A. thaliana* was used to generate stable transgenic plants, the *N. benthamiana* for transient expression assays, and the *B. juncea* for constructing a cDNA library and for endogenous gene expression assays.

### Culture of *B. cinerea*, inoculation, and phenotyping

*B. cinerea* was cultured on potato agar media plus 1.5% dextrose (potato 200g l^−1^, glucose 20g l^−1^, agar 15g l^−1^, pH 6.0) at 22°C for 10 days. The conidia of well-grown *B. cinerea* were suspended in sterile distilled water, filtered with two layers of gauze, and diluted to 5×10^5^ cells per millilitre. Four-week-old *B. juncea* plants and T_2_ transgenic *A. thaliana* plants were inoculated with the conidial suspension by spraying. Distilled water was used as a negative control. Disease phenotyping was performed based on *B. cinerea* biomass quantified by quantitative PCR with *B. cinerea*-specific internal transcribed spacer (ITS) primers Bc-ITS-F/Bc-ITS-R (Table 1).

**Table 1.** The primers used in this study

**Name**	**Sequence (from 5′ to 3′**)	**Feature (direction/role**)
Bait1F	TAAAGCTTCTCTGCTAGAGATAGTGTG	Forward, PCR of Bait and Bait-m
Bait1R	TAGGATCCGTTTCTCTGAGCTGTATGGTTG	Reverse, PCR of Bait and Bait-m
pAbAi-Seq1	GTTCCTTATATGTAGCTTTCGACAT	Forward, sequencing plasmids of pBait-AbAi and pBait-m-AbAi
pAbAi-Seq2	CATGTTAGGATGGGCAAGGCATTGA	Reverse, sequencing plasmids of pBait-AbAi and pBait-m-AbAi
pGADT7-F	TAATACGACTCACTATAGGGC	Forward, sequencing plasmid pGADT7-BjcDNA
pGADT7-R	CTGTGCATCGTGCACCATCT	Reverse, sequencing plasmid pGADT7-BjcDNA
BjMYB1-F1	CGGAATTCATGGGAGTGAAAGGCCTCACC	Forward, PCR *BjMYB1* for construct plasmid pET28a-BjMYB1
BjMYB1-F2	CGGGATCCATGGGAGTGAAAGGCCTCACC	Forward, PCR *BjMYB1* for construct plasmid
		pCAMBIA1307-BjMYB1
BjMYB1-R	GCGTCGACTTATCCAATGGTACTACTAGG	Reverse, PCR *BjMYB1* for construct plasmids
		pET28a-BjMYB1 and pCAMBIA1307-BjMYB1
BjMYB1-F3	AGCTAGGGAAGAGCTATCAG	Forward, RNA quantification of *BjMYB1*
BjMYB1-R2	GAGAGCTTTCAACCGAACAG	Reverse, RNA quantification of *BjMYB1*
BjCHI1-F1	GCACCCGATGGAGCAAATACA	Forward, RNA quantification of *BjCHI1*
BjCHI1-R1	ATTGGTCCTCGTCCGTAGTAA	Reverse, RNA quantification of *BjCHI1*
AtACTIN-F	AGTGGTCGTACAACCGGTATTGT	Forward, for internal reference of *BjMYB1* in *A. thaliana*
AtACTIN-R	GAGGAAGAGCATTCCCCTCGTA	Reverse, for internal reference of *BjMYB1* in *A. thaliana*
BjActin-F	CTTCTTACCGAGGCTCCTCT	Forward, for internal reference of *BjMYB1* and *BjCHI1* in *B. juncea*
BjActin-R	AAGGATCTTCATGAGGTAATCAGT	Reverse, for internal reference of *BjMYB1* and *BjCHI1* in *B. juncea*
Bc-ITS-F	TCGAATCTTTGAACGCACATTGCGC	Forward, for *B. cinerea* biomass quantification
Bc-ITS-R	TGGCAGAAGCACACCGAGAACCTG	Reverse, for *B. cinerea* biomass quantification
Bc-actinF	GAGAGCGGTGGTATCCACGTCAC	Forward, for internal reference of Bc-ITS
Bc-actinR	CACTTGCGGTGGACAATGGAAGGT	Reverse, for internal reference of Bc-ITS

### Yeast one-hybrid assay to screen TFs binding to the Wbl-4 element

**Fig. 1.**
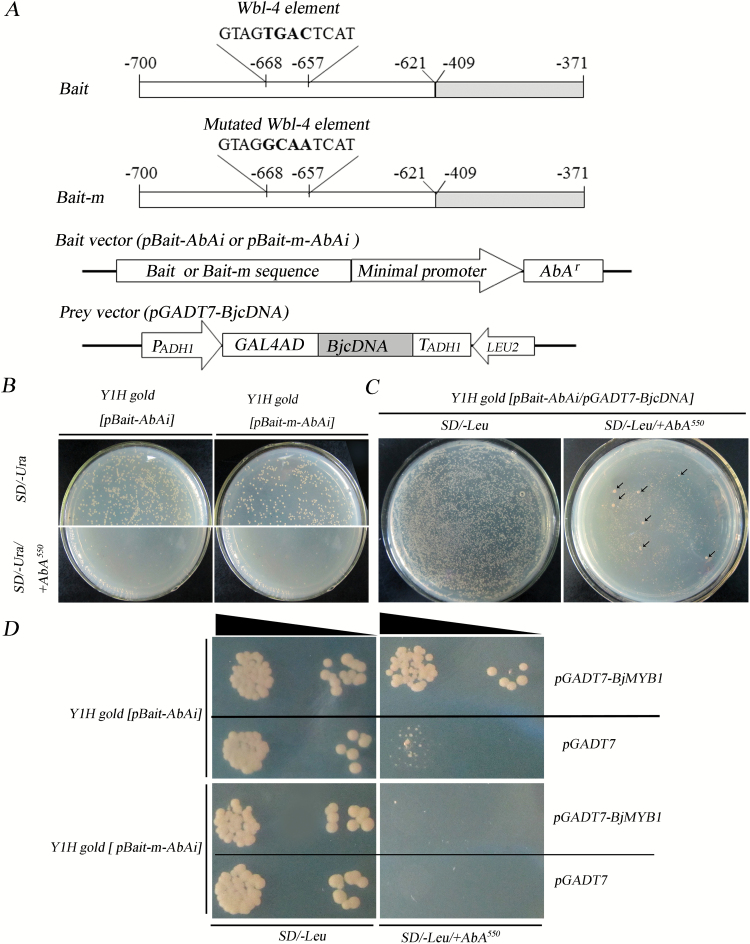
Yeast one-hybrid screening for factors binding to the Wbl-4 element. (**A**) Schematic diagrams of the Bait and Bait-m fragments, and Bait and Prey vectors. The BjC-P promoter fragments -700 to -621 (white box) and -409 to -371 (grey box) were fused as the bait sequence. The numbers above the boxes indicate the nucleotide positions in the BjC-P promoter. The nucleotides in the Wbl-4 element and its mutant are called out wherein the core sequence TGAC and its mutant GCAA are shown in bold. The Bait and Bait-m fragments were, respectively, inserted upstream of the *AbA* ^*r*^ reporter gene in the pBait-AbAi vector. The cDNA from *B. juncea* was inserted into the pGADT7 vector and fused in-frame with *GAL4AD* when preparing the cDNA library. (**B**) Determination of the minimal inhibitory concentration of AbA by growing the bait-reporter yeast strains on the SD/−Ura media with or without AbA. Images show that 550ng ml^−1^ (AbA) was the appropriate inhibitory concentration for the reporter strains Y1Hgold [pBait-AbAi] and Y1Hgold [pBait-m-AbAi]. (**C**) Y1H screening of the *B. juncea* cDNA library. pGADT7-BjcDNA was transferred into the bait-reporter yeast strain Y1Hgold [pBait-AbAi] and then selected on SD/−Leu agar plates containing 550ng ml^−1^ AbA (SD/−Leu/+AbA^550^), using the SD/−Leu agar plate as a control. Arrows indicate the positive clones. (**D**) BjMYB1 interacts with the Wbl-4 element (Bait), but not the mutated Wbl-4 element (Bait-m). The plasmid pGADT7-BjMYB1 isolated from one of the positive clones in (B) was re-transferred into the bait-reporter yeast strains Y1Hgold [pBait-AbAi] and Y1Hgold [pBait-m-AbAi], respectively, and then selected on SD/−Leu/+AbA^550^ agar plates. The transformants from the combination Y1Hgold [pBait-AbAi/pGADT7-BjMYB1] could grow healthily on the SD/−Leu/+AbA^550^ but those from the combination Y1H gold [pBait-m-AbAi/pGADT7-BjMYB1] could not. The empty plasmid pGADT7 and the SD/−Leu agar plate (without AbA) were used as controls.

The pathogen-associated molecular patterns of chitin have been intensively studied (Wan *et al.*, 2012). The water-soluble chitin oligomer hexa-N-acetylchitohexaose can be used as a fungal elicitor in research on plant defence against fungi (Raventos *et al.*, 1995; Chen *et al.*, 2010). To avoid the interference of cDNA from *B. cinerea* in yeast one-hybrid (Y1H) screening, we used this fungal elicitor to treat the *B. juncea* seedling for cDNA library construction. According to our previous study, spraying of 200 µg ml^−1^ hexa-N-acetylchitohexaose solution activates expression of the chitinase gene *BjCHI1* (Gao *et al.*, 2014). Thus, leaves of *B. juncea* seedlings were sprayed with 200 µg ml^−1^ hexa-N-acetylchitohexaose in this study. The sprayed leaves were harvested at 12h, 24h, 48h, and 72h post spraying. Total RNA was extracted from mixture of the harvested leaves for construction of the cDNA library. The *B. juncea* cDNA was cloned into the *Sfi* I site of the pGADT7 vector and fused in-frame with *GAL4AD* (Clontech Laboratories, Inc., a Takara Bio Company), resulting in the cDNA library plasmids, that is, the prey plasmids (Fig. 1A).

The BjC-P promoter fragment (-700 to -621) containing the Wbl-4 and the fragment (−409 to −371), coupled for full magnitude of BjC-P fungal induction (Gao *et al.*, 2014), were conjoined as the Bait sequence for Y1H (Fig. 1A). To obtain genuine positive clones that specifically interact with the Wbl-4 element, a mutant Bait (designated Bait-m) was generated as a negative control, by mutating the core nucleotide sequence TGAC in the Wbl-4 element into GCAA (Fig. 1A). The Bait and Bait-m DNA sequences (see Supplementary Table S1 at *JXB* online) were PCR-amplified from the plasmids P16 and P54 (Gao *et al.*, 2014), respectively, using the primers Bait1F and Bait1R (Table 1). The Bait vectors pBait-AbAi and pBait-m-AbAi were constructed by cloning the Bait or Bait-m sequence into upstream of the *AbA* ^*r*^ reporter gene in the pAbAi vector (Clontech Laboratories, Inc., a Takara Bio Company; Fig. 1A). The constructed pBait-AbAi and pBait-m-AbAi plasmids were then linearized with *BstB* I, and homogenously integrated individually into the chromosome of yeast strain Y1Hgold, resulting in the bait-reporter yeast strains Y1Hgold [pBait-AbAi] and Y1Hgold [pBait-m-AbAi] that were later used to screen the *B. juncea* cDNA library. pBait-AbAi integrations were confirmed by PCR with primers pAbAi-Seq1 and pAbAi-Seq2 (Table 1). Y1H screening experiments were conducted using the Matchmaker Gold Yeast One-hybrid Library Screening System (Clontech Laboratories, Inc., a Takara Bio Company) according to the supplied manual. Y1H positive clones were sequenced with the primers PGADT7-F and PGADT7-R (Table 1).

### Bioinformatic analyses of *BjMYB1*

The software GENSCAN (http://genes.mit.edu/GENSCAN.html, accessed 17 June 2016) was used to predict the open reading frame of *BjMYB1* and the deduced amino acid sequence. The NCBI (http://www.ncbi.nlm.nih.gov/, accessed 17 June 2016) protein domain software was used to analyse the conservative structure domain. NCBI BLAST tools (http://www.ncbi.nlm.nih.gov/, accessed 17 June 2016) were adopted to analyse the orthologs of BjMYB1. Clustalx 1.83 and MEGA 6 were used to make the identity comparison and construct the evolutionary tree, respectively.

### Subcellular localization of BjMYB1

The coding region of *BjMYB1* with the stop codon was inserted into the pCAMBIA1205-YFP vector (Gao *et al.*, 2008) using the *Bam*H I and *Sal* I restriction sites and fused in-frame with the yellow fluorescent protein (YFP) gene under control of the *Cauliflower mosaic virus* (*CaMV*) 35S promoter. The YFP-BjMYB1 fusion protein was transiently expressed in *N. benthamiana* leaves by agroinfiltration described previously (Gao *et al.*, 2014). About 48h post infiltration, the infiltrated *N. benthamiana* leaves were stained with DAPI for about 3h in the dark and then the YFP fluorescence was observed under 514nm excitation, using a confocal laser scanning microscope (Zeiss LSM 700, Germany) with a Fluar ×10/0.50 M27 objective lens and a SP640 filter.

### Protein expression in *Escherichia coli* and EMSA

The vector pET-28a (+) [EMD Biosciences (Novagen), USA] harbouring a His tag was used for *BjMYB1* expression in *Escherichia coli*. *BjMYB1* was cloned into the pET-28a (+) vector by the *EcoR* I and *Sal* I restriction sites. Sequences of primers BjMYB1-F1 and BjMYB1-R used for this cloning were shown in Table 1. The resultant plasmid was transformed into *E. coli* Rosetta (DE3) for expression of the His-BjMYB1 fusion protein. Overexpression of His-BjMYB1 was induced by 0.6mM isopropyl-D-thiogalactoside when the optical density of the bacteria cell culture at 600nm (OD600) reached 0.8. The cells were grown at 16°C overnight, harvested, and homogenized in a buffer containing 50mM NaH_2_PO_4_, 300mM NaCl and 10mM imidazole (pH 8.0). After sonication and centrifugation, the supernatant was applied to Ni^2+^ affinity resin (Ni-NTA, QIAGEN) as described in the manufacturer’s manual. The purified His-BjMYB1 was used for the EMSA.

The EMSA was performed as described previously (Wang *et al.*, 2015). To identify the binding site of BjMYB1, complementary pairs of non-labelled and 3′-biotin-labelled oligonucleotides of BjC-P fragments containing the Wbl-4 or its mutants were synthesized and annealed to generate the double-stranded DNA fragments. Sequences of the synthesized oligonucleotides of W4 (Wbl-4 element intact), W4-d1 (core sequence TGAC in the Wbl-4 element deleted), W4-d2 (central sequence GTGACT of Wbl-4 changed into the typical W-box element TTGACC), W4-d3 (GTGACT sequence of Wbl-4 changed to the typical AC element sequence ACCTACCA), and PAL2Pro (typical AC element in the promoter of the *Phaseolus vulgaris PAL2*) are shown in Supplementary Table S1. The binding reactions were performed in a 20 μl reaction mixture containing 1× binding buffer (Pierce, Rockford, IL, USA), 5mM MgCl_2_, 50ng μl^−1^ poly (dI-dC), 0.05% NP-40 (v/v), 2.5% glycerol (v/v), 40fmol biotin-labelled DNA, 0 or 4 pmol unlabelled DNA, and 15fmol His-BjMYB1 fusion protein. The binding reactions were kept at room temperature for 30min for competition assays, and for another 30min after biotin-labelled DNA was added. Binding reactions were electrophoresed in 8% native polyacrylamide gel and then electrotransferred onto nylon membrane. The transferred DNA was cross-linked to the membrane at 120 mJ cm^−2^ for 1min using a CL-1000 Ultraviolet Crosslinker (UVP, LLC, Upland, CA, USA). Immunostaining was performed with the Light ShiftR Chemiluminescent EMSA Kit (Pierce, Rockford, IL, USA) according to the manufacturer’s protocol.

### Transient expression assays in tobacco leaves

*N. benthamiana* plants were grown until the sixth leaf fully expanded. *Agrobacterium tumefaciens* EHA105 harbouring the construct to be tested was grown on agar-lysogeny broth containing 50 µg ml^−1^ kanamycin and 30 µg ml^−1^ rifampicin, then suspended with MMA buffer (10mM MgCl_2_, 10mM MES pH 5.5, 100 µM acetosyringone) at an OD600 of 0.5, and incubated at 28°C for 3h. The fifth and sixth expanded leaves of *N. benthamiana* were infiltrated with *A. tumefasciens* EHA105 containing the construct to be tested at the blade back with a 1ml needless syringe. Infiltrated leaves were harvested for GUS staining, GUS quantitative assays, or confocal imaging of YFP-BjMYB1 at 48h post inoculation. GUS staining and GUS quantitative assays were performed as described previously (Gao *et al.*, 2014).

### Overexpression of *BjMYB1* in *A. thaliana*

*BjMYB1* cDNA was amplified by RT-PCR, confirmed by sequencing, and then cloned into pCAMBIA1307 at *BamH* I and *Sal* I sites under the control of the 35S promoter. Primers BjMYB1-F2 and BjMYB1-R (Table 1) were used for this cloning. The resultant plasmids were introduced into *A. thaliana* plants through *A. tumefasciens* EHA105 using the floral dip method (Clough and Bent, 1998). Positive transgenic lines were identified by PCR. Seedlings of three independent T_2_ transgenic lines growing in Murashige and Skoog medium with 40 µg ml^−1^ hygromycin were transferred to soil.

### Quantitative real-time PCR

Quantitative real-time (qRT-PCR) was performed using SYBR Premix Ex Taq^TM^ II (TAKARA BIO INC, Japan) and a 7500 Fast Real-Time PCR System (Applied Biosystems, Foster City, CA, USA) with the following conditions: 95°C for 1min, 40 cycles of 95°C for 10s, and 60°C for 34s in 20 μl reaction volumes. A dissociation curve was generated for each reaction to ensure specific amplification. The actin genes of *A. thaliana*, *B. juncea*, and *B. cinerea* were used as their own internal references. The relative expression was quantified using the comparative 2^−ΔΔCT^ method (Livak and Schmittgen, 2001). Primer pairs BjMYB1-F3/BjMYB1-R2, BjCHI1-F1/BjCHI1-R1, and Bc-ITS-F/Bc-ITS-R (Table 1) were used for qRT-PCR analysis of *BjMYB1*, *BjCHI1*, and *Bc-ITS*, respectively. Primer pairs of the corresponding internal reference genes, AtACTIN-F/AtACTIN-R, BjActin-F/BjActin-R, and Bc-actinF/Bc-actinR, are also shown in Table 1.

## Results

### Molecular cloning of *BjMYB1*

Our previous work revealed that the Wbl-4 motif (GTAGTGACTCAT) is the core fungus-responsive *cis*-acting element in BjC-P, the promoter of the unusual chitinase gene *BjCHI1* (Gao *et al.*, 2014). To identify TFs that interact with the *cis*-acting element Wbl-4, we performed Y1H screening of a *B. juncea* cDNA library. The Bait fragments and Bait and Prey vectors used for the Y1H screening are schematically shown in Fig. 1A.

To perform the Y1H screening, the minimal inhibitory concentration of aureobasidin A (AbA) for the bait-reporter yeast strains Y1Hgold [pBait-AbAi] and Y1Hgold [pBait-m-AbAi] were first determined by growing them on the SD/−Ura/+AbA media, resulting in an appropriate inhibitory concentration of 550ng ml^−1^ AbA (Fig. 1B). The pGADT7-BjcDNA plasmids (1 µg) from the *B. juncea* cDNA library were then used to transform the bait-reporter yeast strain Y1Hgold [pBait-AbAi]. Transformants were cultured on SD/−Leu (as a control) and SD/−Leu/+AbA^550^ (SD/−Leu media containing 550ng ml^−1^ AbA) agar plates (Fig. 1C). The single-cell clones growing healthy on the SD/−Leu/+AbA^550^ agar plate were recognized as positive clones. The pGADT7-BjcDNA plasmids in each of the positive clones were isolated and further transformed into the bait-reporter strain Y1Hgold [pBait-m-AbAi], and transformants were screened on the same SD/−Leu/+AbA^550^ media. Among the pGADT7-BjcDNA plasmids from 28 positive clones, seven could not generate Y1Hgold [pBait-m-AbAi] transformants, suggesting that the inserted BjcDNA in the seven pGADT7-BjcDNA plasmids encode proteins specifically binding to the Wbl-4 element. Sequencing and BLAST analysis revealed that three of the seven pGADT7-BjcDNA plasmids harbour an identical BjcDNA that encodes a putative MYB protein. Because no uniform nomenclature has been proposed for MYB-type genes from *B. juncea*, we designated the BjcDNA as *BjMYB1* (Fig. 1D).

### *BjMYB1* encodes an R2R3-MYB protein located in the plant cell nucleus

**Fig. 2.**
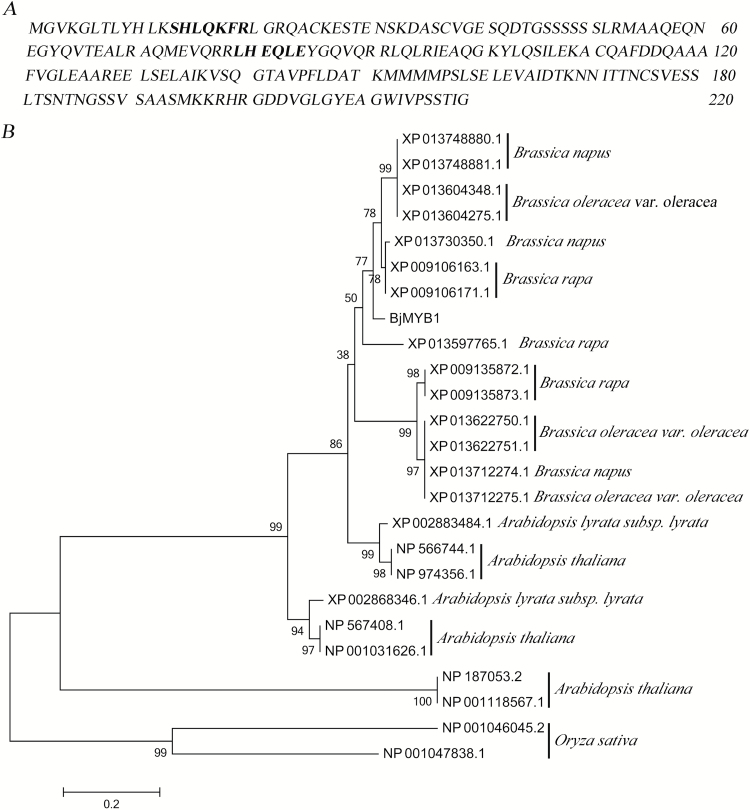
Amino acid sequence of BjMYB1 and phylogenetic analysis. (**A**) The deduced 220 amino acids and the putative two MYB domains (in bold) of BjMYB1. (**B**) Phylogenetic analysis of BjMYB1 with the orthologs from *Brassica*, *Arabidopsis*, and *Oryza*. The software Clustalx 1.83 and MEGA 6 were used to make the identity comparison and construct the evolutionary tree, respectively. Node values are percentages of bootstraps generated with 1000 bootstrap replicates. The bar shows an evolutionary distance corresponding to 0.2 amino acid substitutions per site.

Bioinformatic analysis revealed that the *BjMYB1* cDNA isolated by the Y1H screening contains a 663-bp open reading frame that encodes a 220-amino acid protein with two MYB domains (SHLQKFR and LHEQLE, Fig. 2A). We used the BjMYB1 protein as a query sequence to BLAST the NCBI database; the searched orthologs of BjMYB1 were annotated as MYB family members, but none of them has been assigned a function experimentally. The orthologs from species *Brassica* (with 100% coverage and >80% identity), *Arabidopsis* (with >80% coverage and >80% identity) and *O*. *sativa* (with >80% coverage) were used for multiple sequence alignments (see Supplementary Fig. S1 at *JXB* online) and maximum-likelihood phylogenetic analysis (Fig. 2B). The phylogenetic analysis allocated BjMYB1 to a distinct subclade, with closest relationship to a cluster containing XP 013730350.1, XP 009106163.1, and XP 009106171.1 from *Brassica* (Fig. 2B). Three MYB family members (NP 566744.1, NP 974356.1, and XP 002883484.1) from *Arabidopsis* are relatively close orthologs of BjMYB1. BjMYB1 showed the most distant phylogenetic relationships with orthologs from *O. sativa* (Fig. 2B and Supplementary Fig. S1).

**Fig. 3.**
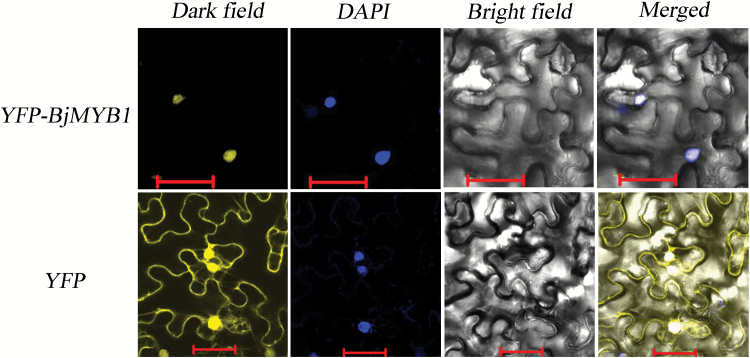
BjMYB1 is localized in the nucleus of *N. benthamiana* cells. Construct pCAMBIA1205-YFP-BjMYB1 was infiltrated into *N. benthamiana* leaves for transient expression of YFP-BjMYB1. pCAMBIA1205-YFP was infiltrated as a control. Images were taken about 48h after infiltration and the infiltrated tobacco leaves were stained with DAPI before the photo was taken. The experiments were repeated at least three times with similar results. YFP indicates yellow fluorescent protein. Bar = 50 µm.

To validate the prediction that BjMYB1 is a TF, we examined its subcellular localization. The chimeric expression vector pCAMBIA1205-YFP-BjMYB1 and the control vector pCAMBIA1205-YFP were constructed and delivered into *N. benthamiana* leaves via agroinfiltration (Wu *et al.*, 2009). Confocal imaging of the transient expression showed that YFP-BjMYB1 accumulated only in the cell nucleus, whereas YFP alone was present throughout the whole cell as expected, indicating that BjMYB1 is a nucleus-localized protein (Fig. 3), consistent with the fact that TFs typically function in the cell nucleus.

### BjMYB1 binds to the Wbl-4 element *in vitro*

**Fig. 4.**
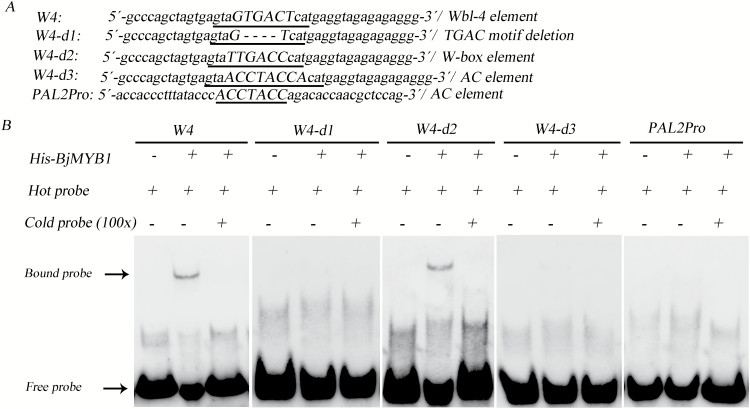
BjMYB1 binds to the Wbl-4 and W-box element *in vitro*. (**A**) Nucleotide sequences of the probes with central nucleotides in bold and underlined. W4 contains the Wbl-4 element. W4-d1 is a mutant of W4 with the core sequence TGAC in the Wbl-4 element deleted (----). W4-d2 is a mutant of W4 with the GTGACT changed into a typical W-box element motif TTGACC. W4-d3 is another mutant of W4 with the GTGACT changed into the AC element motif ACCTACCA. PAL2Pro is the promoter fragment of the bean *PAL2* gene containing the AC element ACCTACC. (**B**) EMSA for the DNA-binding activity of the His-BjMYB1 fusion protein with W4, W4-d1, W4-d2, W4-d3, and PAL2Pro probes. An equal amount of BjMYB1 protein or hot probe (biotin-labelled) was used in all lanes. There was 100 times the amount of cold probes (without the biotin label) than hot probes for competitive binding. The bound and free hot probes are indicated by arrows on the left.

To confirm the binding of BjMYB1 to the Wbl-4 element (GTAGTGACTCAT), a His-tagged BjMYB1 fusion protein was expressed in *E. coli* Rosetta (DE3) (see Supplementary Fig. S2 at *JXB* online) and used for an EMSA. An obviously shifted band was observed in the assay with the Wbl-4 element (Fig. 4, W4), indicating the binding between BjMYB1 and the Wbl-4 element. Consistently, when the central sequence GTGACT of Wbl-4 was changed into the typical W-box element TTGACC, an obviously shifted band still appeared (Fig. 4, W4-d2). Conversely, when the core sequence TGAC in the Wbl-4 element was deleted, the shifted band disappeared, indicating that the mutation of the core sequence TGAC abolished the binding between BjMYB1 and the Wbl-4 element (Fig. 4, W4-d1). These results indicate that the isolated BjMYB1 indeed interacts with the Wbl-4 element and that the TGAC core element is essential for binding of BjMYB1 to Wbl-4.

Because AC elements are the known binding target of some R2R3-MYB proteins (Prouse and Campbell, 2012), we then examined the ability of BjMYB1 to bind to AC elements by changing the GTGACT sequence of Wbl-4 into the typical AC element sequence ACCTACCA (Fig. 4A, W4-d3). EMSA assays showed that BjMYB1 displayed almost no binding affinity to this AC element generated from the Wbl-4 element (Fig. 4B, W4-d3). This observation was further confirmed by EMSA assays with the typical AC element in the promoter of the *P*. *vulgaris PAL2* gene (Patzlaff *et al.*, 2003*a*) (Fig. 4, PAL2Pro). PAL2Pro contains the AC element, which is the binding target of the R2R3-MYB protein PtMYB4 (Patzlaff *et al.*, 2003*a*). These results clearly showed that the R2R3-MYB protein BjMYB1 could bind to W-box or Wbl elements, but not to the AC elements tested.

### BjMYB1 activates promoter BjC-P by binding to the Wbl-4 element *in vivo*

**Fig. 5.**
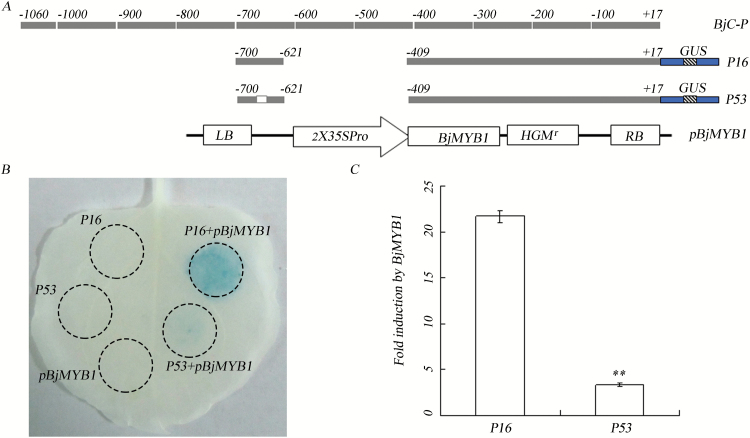
BjMYB1 activates BjC-P by binding to the Wbl-4 element *in vivo*. (**A**) Schematic diagram of BjC-P, GUS-expressing constructs P16, P53, and the *BjMYB1*-expressing construct p*BjMYB1*. Grey boxes represent BjC-P and its deletion derivatives. The numbers above the boxes indicate the nucleotide positions relative to the *BjCHI1* transcription start site. Blue boxes represent the *GUS* gene containing an intron indicated by the texture area. The small white box in P53 indicates deletion of the core sequence TGAC in Wbl-4. (**B**) GUS histochemical staining of a tobacco leave infiltrated or co-infiltrated with the constructs as indicated. The dashed circles indicate the infiltration areas. (**C**) Quantization of GUS activity of P16 and P53 in transiently transformed tobacco leaves. *N. benthamiana* leaves were co-infiltrated with P16 or P53 and p*BjMYB1* as well as a *CaMV* 35S::LUCint construct (pBI121-LUCint). Each of the plasmids was adjusted into equal density for the infiltrations. The GUS activity was normalized with the LUC activity. Columns show the ratio of GUS activity induced by p*BjMYB1* to that of no induction. Values represent means ± SD from three replicates. The asterisks indicate significant difference (one-tail *t*-test, compared with control, *P* < 0.01).

To validate the transcriptional activation of the promoter BjC-P by BjMYB1, we performed transactivation analysis by transient expression in *N. benthamiana*. Previously, we revealed that the BjC-P deletion derivative P16 containing the Wbl-4 element was sufficient for fungus-response, and this response was abolished by deletion of the core sequence TGAC in the Wbl-4 element as represented by the deletion derivative P53 (Gao *et al.*, 2014). Therefore, we used P16 and its mutant P53 as reporter plasmids (Fig. 5A). To express BjMYB1 *in planta*, we constructed the plasmid p*BjMYB1* by placing the *BjMYB1* cDNA downstream of the 35S promoter in the binary vector pCAMBIA1307 (Lin *et al.*, 2014). In the transactivation analysis, *N. benthamiana* leaves were co-infiltrated with the construct combinations P16/p*BjMYB1* and P53/p*BjMYB1*; the construct P16 or P53 or p*BjMYB1* alone were infiltrated as negative controls. In addition, the pBI121-LUCint, a *CaMV* 35S::LUCint construct, was co-infiltrated in each assay to normalize the GUS activity by its LUC activity (Wu *et al.*, 2009; Gao *et al.*, 2014). GUS staining and GUS quantitative assays of the infiltrated leaves showed that overexpression of *BjMYB1* strongly activated the BjC-P promoter derivative P16, but not the mutant P53 (Fig. 5B, C). Given that P53 lacks only the core sequence TGAC in the Wbl-4 element compared with P16, these observations indicate that BjMYB1 could specifically recognize the Wbl-4 element *in vivo*, consistent with the results of the Y1H and the EMSA assays. Taken together, aforementioned experiments demonstrate that BjMYB1 functions as a transcription activator by interacting with the Wbl-4 element of BjC-P for target gene activation.

### BjMYB1 is involved in positive regulation of plant defence against *B. cinerea*

**Fig. 6.**
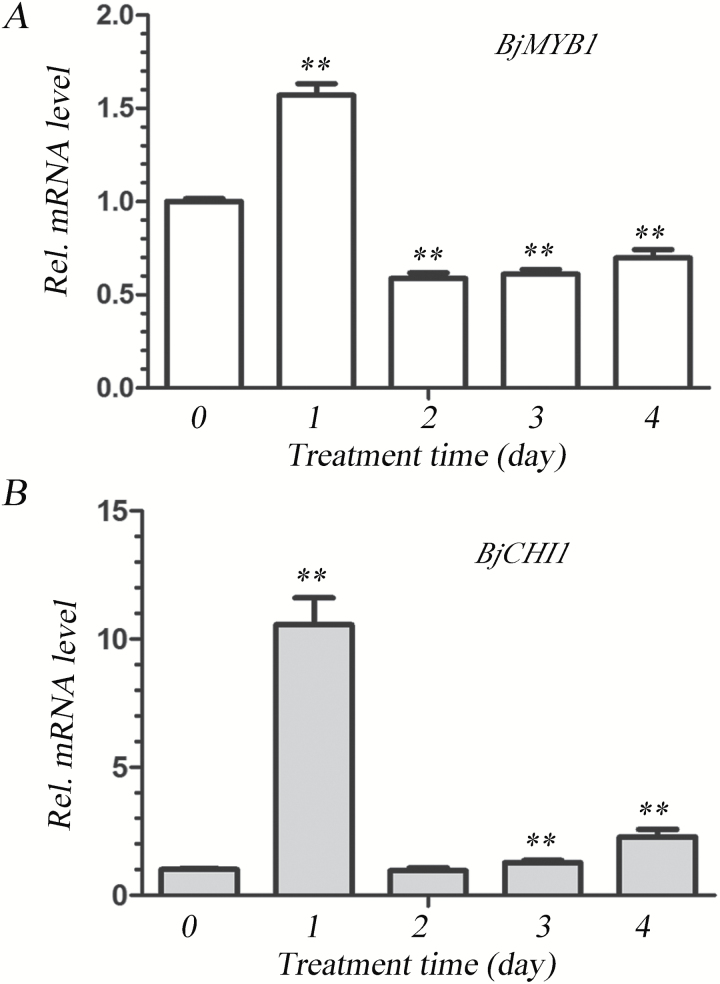
mRNA expression of *BjMYB1* (**A**) and *BjCHI1* (**B**) in native host plant *B. juncea* responding to infection by *B. cinerea*. Leaves of *B. juncea* seedlings were inoculated with *B. cinerea* and harvested at 1 d, 2 d, 3 d, and 4 d post inoculation, respectively. Total RNAs were extracted for qPCR analysis. The actin gene of *B. juncea* was used as an internal control. All real-time results were normalized with respect to the expression level of that at 0 d. The data are shown as mean values ± SD from three replicates. The asterisks indicate significant difference (two-tail *t*-test, compared with control, *P* < 0.01).

It has been shown that the two-chitin-binding-domains chitinase BjCHI1 has anti-fungal activities against *B. cinerea* (Guan and Chye, 2008; Guan *et al.*, 2008). To investigate the function of BjMYB1 and its association with BjCHI1 in the regulation of plant defence against *B. cinerea*, we examined the expression levels of *BjMYB1* and *BjCHI1* in their native host *B. juncea* upon infection by *B. cinerea*. qPCR analysis showed that the transcription of *BjMYB1* was strongly induced by *B. cinerea*, reaching a peak at 1 day post inoculation (Fig. 6A). The expression pattern of *BjMYB1* was similar to that of *BjCHI1* (Fig. 6B). This result along with the aforementioned *in vivo* BjC-P promoter activation results suggests that BjMYB1 is potentially involved in host defence against fungal attack by activating the expression of *BjCHI1* by binding to the Wbl-4 element in the BjC-P promoter.

**Fig. 7.**
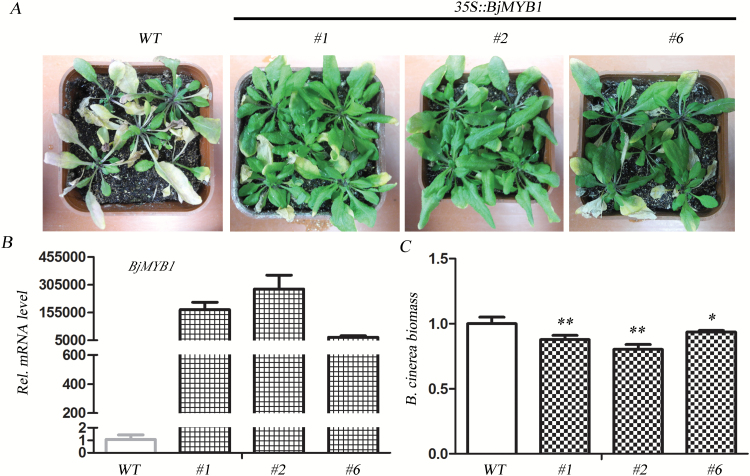
*BjMYB1*-overexpressing *A. thaliana* plants exhibit enhanced disease resistance to *B. cinerea*. (**A**) Phenotypes of the wild-type Col-0 (WT) and three *BjMYB1*-overexpressing *A. thaliana* lines (#1, #2, and #6) at 3 weeks post inoculation with *B. cinerea*. (**B**) qPCR analysis on *BjMYB1* expression in transgenic *A. thaliana* lines inoculated with *B. cinerea*. The real-time results were normalized with respect to the expression level in wild-type Col-0. (**C**) The biomass of *B. cinerea* in wild-type Col-0 and *BjMYB1*-overexpressing *A. thaliana* leaves inoculated with *B. cinerea* was measured by qPCR. The asterisks indicate significant difference (one-tail *t*-test, compared with WT, **P < 0.01; *P < 0.05).

To further evaluate the function of BjMYB1 in plant defence against *B. cinerea*, we generated stable transgenic *A. thaliana* plants overexpressing *BjMYB1* by *Agrobacterium*-mediated transformation. Twelve independent transgenic plants were generated and three homozygous T_2_ lines were used for intensive assays. Seedlings of the transgenic *A. thaliana* were inoculated with *B. cinerea*, and the disease was assessed by disease severity and *B. cinerea* biomass in inoculated leaves (Veronese *et al.*, 2006; Weiberg *et al.*, 2013). As shown in Fig. 7, serious disease symptoms appeared on the leaves of the wild type at 3 weeks post inoculation (wpi) (Fig. 7A). In contrast, the transgenic *A. thaliana* plants overexpressing *BjMYB1* were relatively healthy at 3 wpi, indicating an enhanced resistance to *B. cinerea* (Fig. 7A). At the mature stage, the leaves of wild type had almost died and the plants produced few pods (see Supplementary Fig. S3 at *JXB* online). In contrast, the leaves of the *BjMYB1*-transgenic *A. thaliana* lines were still green and healthy and the plants produced lots of pods (Supplementary Fig. S3). In concordance with the disease phenotypes, the disease-resistant *BjMYB1*-expressing transgenic lines showed higher mRNA levels of *BjMYB1* (Fig. 7B) and lower *B. cinerea* biomass compared with the wild type (Fig. 7C). These results demonstrate that BjMYB1 plays a positive role in plant defence against *B. cinerea*.

## Discussion

Understanding plant defences at molecular level will help breeders to utilize the resistance mechanisms in crop improvement. Based on our previous findings that the *BjCHI1* promoter BjC-P is multi-stress responsive (Wu *et al.*, 2009) and that Wbl-4 is the core element responsive to *B. cinerea* infection (Gao *et al.*, 2014), we have carried out further analysis on the molecular mechanism of BjC-P-mediated fungal resistance. We have isolated an R2R3-MYB TF, BjMYB1, from *B. juncea* by Y1H screening and shown that BjMYB1 binds to the Wbl-4 element *in vitro*, as well as that overexpression of *BjMYB1* can activate the BjC-P promoter by acting with the Wbl-4 element *in vivo*. Deletion of the core sequence TGAC in the Wbl-4 element completely abolished the interaction between BjMYB1 and the Wbl-4 in both yeast and tobacco. In addition, the *B. cinerea*-induced mRNA expression pattern of *BjMYB1* was similar to that of *BjCHI1* in the native host plant *B. juncea*. Overexpression of *BjMYB1* in stable, transgenic *A. thaliana* plants enhanced host plant resistance to *B. cinerea*. These results suggest that BjMYB1 might be involved in plant defence against fungal infection by interacting with the Wbl-4 element and regulating the expression of *BjCHI1* for host plant defence.

Previous investigations have revealed that W-box element (T/C)TGAC(C/T) or Wbl elements (containing a core motif TGAC) are recognized by WRKY transcriptional factors (Eulgem *et al.*, 2000; Eulgem and Somssich, 2007; Rushton *et al.*, 2010; Chi *et al.*, 2013; Lu *et al.*, 2013). In this study, our results showed that the isolated BjMYB1, an R2R3-type MYB transcriptional factor, could bind to the Wbl-4 element. However, most reported DNA targets of plant R2R3-MYB TFs are AC elements (Grotewold *et al.*, 1994; Patzlaff *et al.*, 2003a, b; Goicoechea *et al.*, 2005; Fornalé *et al.*, 2010; Prouse and Campbell, 2012, 2013). To our surprise, when the GTGACT sequence of Wbl-4 was changed into the typical W-box element (TTGACC), BjMYB1 still showed strong binding ability (Fig. 4, W4-d2), but very faint binding affinity to the AC element in W4-d3 (Fig. 4, W4-d3) and no binding affinity to the typical AC element in the PAL2Pro probe (Fig. 4, PAL2Pro). This is consistent with the recent finding that R2R3-MYBs can recognize a variety of DNA motifs and the binding specificities are relevant to their biological roles (Kelemen *et al.*, 2015). Further, Wang and colleagues have demonstrated that the RAV (TaRAV) protein, an AP2/ERF and B3 domain-containing TF, can bind to a W-box motif (CTGACT) in the promoter of *TaeIF5A1* (Wang *et al.*, 2012). Based on these emerging factors, it seems conceivable that BjMYB1 binds to the Wbl-4 element to regulate plant defence against fungus.

We also examined the interactions between BjMYB1 and another five Wbl elements in the BjC-P by EMSA assays and found that BjMYB1 also showed faint binding to Wbl-1, Wbl-3, and Wbl-6, but not to Wbl-2 or Wbl-5 (see Supplementary Fig. S4 at *JXB* online); this variation might be conferred by the delicate differences in the sequences flanking the core motif (TGAC) in the six Wbl elements. Ciolkowski *et al.* (2008) and Brand *et al.* (2010) also found that additional nucleotide sequences flanking W-box elements conferred a certain level of specificity of binding. Based on our previous finding that BjC-P is a multi-stress inducible promoter, responsive not only to fungal attack but also to abiotic stresses such as salt, drought, and wounding (Wu *et al.*, 2009), we surmise that BjMYB1 might also be involved in responses to abiotic stresses by interacting with some of the Wbl elements. Furthermore, W-box is involved in the regulation of NaCl-inducible expression of *At*WRKY25 and *At*WRKY33 (Jiang and Deyholos, 2009) and many *Botrytis*-induced genes, such as the BOS1 R2R3MYB TF protein in *Arabidopsis* that is required for biotic and abiotic stress responses (AbuQamar *et al.*, 2006; Mengiste *et al.*, 2003). It is evident that our present data cannot fully explain *in vivo* functional binding of BjMYB1 to the Wbl elements. Nonetheless, our results provide important information that it is the coupling of the flanking sequence around the W-box core motif (TGAC) that defines a specific functional element.

The ectopic overexpression of *BjMYB1* in heterologous *A. thaliana* strengthened its resistance to *B. cinerea*. However, it is a pity that our present data cannot fully elucidate the impact of BjMYB1 expression on the native regulatory networks. It is known that plant resistance to *B. cinerea* is determined by multiple host and environmental factors. In contrast to responses to biotrophic pathogens, which are governed by gene-for-gene interactions, resistance to *B. cinerea* likely requires contributions from multiple genes for full resistance (Stracke *et al.*, 2001; Windram *et al.*, 2012). Plant resistance to *B. cinerea* likely involves a complex genetic network which requires plant hormone synthesis and signalling, removal of reactive oxygen species, and biotic and abiotic stress responses (AbuQamar *et al.*, 2006); indeed, Ma and colleagues reported that some genes containing the same motifs could form expression modules in a co-expression network depending on their expression patterns (Ma *et al.*, 2013). For instance, the module I genes of the MYB TF family regulated by the MYB motif CCwACC (with ‘w’ standing for ‘A’ or ‘C’) are involved in the response to the fungal pathogen *B. cinerea* in a co-expression network (Ma *et al.*, 2013). Thus, whether or not other regulatory factor(s) are coupled with BjMYB1 to co-defend against *B. cinerea* infection is still unknown. A phylogram analysis showed that *A. thaliana* harbours BjMYB1 orthologs that would presumably target similar promoters, implying the possibility that BjMYB1 interacts or competes with endogenous MYBs or even WRKYs, resulting in the resistance of the *BjMYB1*-overexpressing transgenic *A. thaliana* plants (Fig. 7).

Finally, BjC-P is a multi-stress inducible promoter which is responsive to treatments of jasmonic acid, fungi, drought, and salt (Wu *et al.*, 2009). Detailed characterization of the signalling pathways associated with BjC-P and BjMYB1 might provide new insights into the development of crops with enhanced tolerance to necrotrophic pathogens.

## Supplementary data

Supplementary data are available at *JXB* online.

**Table S1.** The oligonucleotide sequences used in this study.

**Fig. S1.** Alignments of amino acid sequences of BjMYB1 and similar MYB-type proteins from *Brassica*, *Arabidopsis*, and *Oryza*.

**Fig. S2.** Expression of His-BjMYB1 fusion protein in *E. coli* Rosetta (DE3).

**Fig. S3.** *BjMYB1*-overexpressing *Arabidopsis* plants exhibited enhanced disease resistance to *B. cinerea* at mature stage.

**Fig. S4.** EMSA analyses for the interaction between BjMYB1 and five other Wbl elements in BjC-P.

Supplementary Data

## Abbreviations:

AbA: aureobasidin A
B. cinerea: *Botrytis cinerea*
B. juncea: *Brassica juncea*
EMSA: electrophoretic mobility shift assay
qRT-PCR: quantitative real-time-PCR
TF: transcription factor
Wbl: W-box-like
Wbl-4: W-box-like-4
wpi: weeks post inoculation.
