# Supplementary material for: BjMYB1, a transcription factor implicated in plant defence through activating BjCHI1 chitinase expression by binding to a W-box-like element
Source: J Exp Bot. 2016 Jun 27;67(15):4647–58. doi: 10.1093/jxb/erw240 (PMC4973735; doi:10.1093/jxb/erw240)
Supplement: Supplementary Data [file supp_67_15_4647__index.html]

BjMYB1, a transcription factor implicated in plant defence through activating BjCHI1 chitinase expression by binding to a W-box-like element — BjMYB1, a transcription factor implicated in plant defence through activating BjCHI1 chitinase expression by binding to a W-box-like element — Supplementary Data 

# BjMYB1, a transcription factor implicated in plant defence through activating *BjCHI1* chitinase expression by binding to a W-box-like element

## Supplementary Data

Data files

- supplementary\_table\_S1\_figures\_S1\_S4.pdf - Supplementary Data
