# Supplementary material for: BjMYB1, a transcription factor implicated in plant defence through activating BjCHI1 chitinase expression by binding to a W-box-like element
Source: J Exp Bot. 2016 Jun 27;67(15):4647–58. doi: 10.1093/jxb/erw240 (PMC4973735; doi:10.1093/jxb/erw240)
Supplement: Supplementary Data [file supp_erw240_supplementary_table_S1_figures_S1_S4.pdf]

Supplementary data for

BjMYB1, a transcription factor implicated in plant defense through activating BjCHI1 chitinase expression by binding to a W box-like element

Ying Gao, Shuangwei Jia, Chunlian Wang, Fujun Wang, Fajun Wang, Kaijun Zhao

Supplemental table and Figures

Table S1. The oligonucleotides sequences used in this study

Name	Sequence (from 5' to 3') and feature	
Bait	CTCTGCTAGAGATAGTGTGGCCCAGCTAGTGAGTAGTGACTCATGAGGTAGAGAGAGGGTGGTC<br>CATCATATATCGTGTTGCATGCGCTAGAGAGAGGGTGGTCCAGCTATCGTGTAGAAATATT	
Bait-m	CTCTGCTAGAGATAGTGTGGCCCAGCTAGTGAGTAGGCAATCATGAGGTAGAGAGAGGGTGGTC<br>CATCATATATCGTGTTGCATGCGCTAGAGAGAGGGTGGTCCAGCTATCGTGTAGAAATATT	
W4-F	GCCCAGCTAGTGAGTAGTGACTCATGAGGTAGAGAGAGGG	
W4-d1F	GCCCAGCTAGTGAGTAGTCATGAGGTAGAGAGAGGG	
W4-d2F	GCCCAGCTAGTGAGTATTGACCCATGAGGTAGAGAGAGGG	
W4-d3F	GCCCAGCTAGTGAGTAACCTACCACATGAGGTAGAGAGAGGG	
PAL2Pro-F	ACCACCCTTTATACCCACCTACCAGACACCAACGCTCCAG	
W1-F	AGTCCAAAATAACAAATGACAATTCCACCTTTATTTTAAC	
W2-F	AGGGTGGTACAGTCAGTGACTCCTGAGCTTCAGAGAGGGT	
W3-F	GTAGTCCAGCAGCTAGTGACTCTGCTAGAGATAGTGTGGT	
W5-F	AGGGTGGTCCCTCTAGTGACTCATGAGCTAGAGAGAGGGT	
W6-F	CAAATGAACTTGGTGGTGACAAAATAGAAGTTTAGAATGT	
W4-RBio	CCCTCTCTCTACCTCATGAGTCACTACTCACTAGCTGGGC	5 ́-biotin-labeled;
W4-d1RBio	CCCTCTCTCTACCTCATGACTACTCACTAGCTGGGC	5 ́-biotin-labeled;
W4-d2RBio	CCCTCTCTCTACCTCATGGGTCAATACTCACTAGCTGGGC	5 ́-biotin-labeled;
W4-d3RBio	CCCTCTCTCTACCTCATGTGGTAGGTTACTCACTAGCTGGGC	5 ́-biotin-labeled;
PAL2Pro-RBio	CTGGAGCGTTGGTGTCTGGTAGGTGGGTATAAAGGGTGGT	5 ́-biotin-labeled;
W1-Rbio	GTTAAAATAAAGGTGGAATTGTCATTTGTTATTTTGGACT	5 ́-biotin-labeled;
W2-Rbio	ACCCTCTCTGAAGCTCAGGAGTCACTGACTGTACCACCCT	5 ́-biotin-labeled;
W3-Rbio	ACCACACTATCTCTAGCAGAGTCACTAGCTGCTGGACTAC	5 ́-biotin-labeled;
W5-Rbio	ACCCTCTCTCTAGCTCATGAGTCACTAGAGGGACCACCCT	5 ́-biotin-labeled;
W6-Rbio	ACATTCTAAACTTCTATTTTGTCACCACCAAGTTCATTTG	5 ́-biotin-labeled;

```
BjMYB1           ..............................................  0
XP_013712274.1   ...mysairs.lpldgg....eyhgpldgtnlpgdaclvlttdpkp 38  85%
XP_013730350.1   ...mfthlge.rerer.....erererdgtnlpgdaclvlttdpkp 37  93%
XP_013748880.1   ...mysairs.lpldgg....dyhgpldgtnlpgdaclvlttdpkp 38  92%
XP_013748881.1   ...mysairs.lpldgg....dyhgpldgtnlpgdaclvlttdpkp 38  91%
XP_013604275.1   ...mysairs.lpldgg....dhhgpldgtnlpgdaclvlttdpkp 38  92%
XP_013622751.1   .....................myhgpldgtnlpgdaclvlttdpkp 25  85%
XP_013604348.1   ...mysairs.lpldgg....dhhgpldgtnlpgdaclvlttdpkp 38  92%
XP_013622750.1   .....................myhgpldgtnlpgdaclvlttdpkp 25  85%
XP_013712275.1   ...mysairs.lpldgg....eyhgpldgtnlpgdaclvlttdpkp 38  84%
XP_009106163.1   ...mysairs.lpldgg....dyhgpldgtnlpgdaclvlttdpkp 38  93%
XP_009106171.1   ...mysairs.lpldgg....dyhgpldgtnlpgdaclvlttdpkp 38  92%
XP_009135872.1   ...mysairs.lpldgg....eyhgpldgtnlpgdaclvlttdpkp 38  85%
XP_009135873.1   ...mysairs.lpldgg....eyhgpldgtnlpgdaclvlttdpkp 38  84%
XP_013597765.1   .......mys.lpldgg....eyhgpldgtdlpgdaclvlttdpkp 34  85%
XP_002868346.1   ...mysaihsslpldg.....nlgeysdgtnlpidaclvlttdpkp 38  77%
XP_002883484.1   ...mysairs.lpldgghaagdyhgpldgtnlpgdaclvlttdpkp 42  88%
NP_187053.2      myyqnqhqgknilsssrmhitserhpflrgnspgdsglilstdakp 46  40%
NP_566744.1      ...mysairs.lpldgghvggdyhgpldgtnlpgdaclvlttdpkp 42  87%
NP_567408.1      ...mysairsslpldg.....slgdysdgtnlpidaclvlttdpkp 38  76%
NP_974356.1      ...mysairs.lpldgghvggdyhgpldgtnlpgdaclvlttdpkp 42  88%
NP_001031626.1   ...mysairsslpldg.....slgdysdgtnlpidaclvlttdpkp 38  77%
NP_001118567.1   myyqnqhqgknilsssrmhitserhpflrgnspgdsglilstdakp 46  43%
NP_001046045.2   .............................mcvqgdsglvlttdpkp 17  42%
NP_001047838.1   ...mfegmer....ag......ygvgvggagavg.agvvlsrdpkp 32  41%

BjMYB1           .................................MGVKGLTLYHLKS 13
XP_013712274.1   rlrwtaelherfveavtelggpekatpktlmrt------------- 84
XP_013730350.1   rlrwtaelherfvdavtqlggpdkatpktimrt------------- 83
XP_013748880.1   rlrwtaelherfvdavtqlggpdkatpktimrt------------- 84
XP_013748881.1   rlrwtaelherfvdavtqlggpdkatpktimrt------------- 84
XP_013604275.1   rlrwtaelherfvdavtqlggpdkatpktimrt------------- 84
XP_013622751.1   rlrwtaelherfveavtelggpekatpktlmrt------------- 71
XP_013604348.1   rlrwtaelherfvdavtqlggpdkatpktimrt------------- 84
XP_013622750.1   rlrwtaelherfveavtelggpekatpktlmrt------------- 71
XP_013712275.1   rlrwtaelherfveavtelggpekatpktlmrt------------- 84
XP_009106163.1   rlrwtaelherfvdavtqlggpdkatpktimrt------------- 84
XP_009106171.1   rlrwtaelherfvdavtqlggpdkatpktimrt------------- 84
XP_009135872.1   rlrwtaelherfveavtelggpekatpktlmrt------------- 84
XP_009135873.1   rlrwtaelherfveavtelggpekatpktlmrt------------- 84
XP_013597765.1   rlrxtaelherfvdavsqlggpdkatprtimrt------------- 80
XP_002868346.1   rlrwtselherfvdavtqlggpdkatpktimrt------------- 84
XP_002883484.1   rlrwttelherfvdavtqlggpdkatpktimrt------------- 88
NP_187053.2      rlkwtpdlherfieavnqlggadkatpktimkv--ip--------- 92
NP_566744.1      rlrwttelherfvdavtqlggpdkatpktimrt------------- 88
NP_567408.1      rlrwtselherfvdavtqlggpdkatpktimrt------------- 84
NP_974356.1      rlrwttelherfvdavtqlggpdkatpktimrt------------- 88
NP_001031626.1   rlrwtselherfvdavtqlggpdkatpktimrt------------- 84
NP_001118567.1   rlkwtpdlherfieavnqlggadkatpktimkv--ip--------- 92
NP_001046045.2   rlrwtvelherfvdavtqlggpdkatpktimrv------------- 63
NP_001047838.1   rlrwtpdlherfveavtklggpdkatpksvlrl--m---------- 78

BjMYB1           HLQKFRLGRQACKES..TENSKDASCVGES.QDTGSSSSSSLRMAA 56
XP_013712274.1   ------q------d-t.dnsn---------.-------p---kl-- 128
XP_013730350.1   -------------d-..-d-----------.--------------- 126
XP_013748880.1   -------------d-..-d-------a---.--------------- 127
XP_013748881.1   -------------d-..-d-------a---.--------....... 120
```

```
XP_013604275.1    -------------d-..-d-------a---.--------------- 127
XP_013622751.1    ------q------d-t.dnsn---------.-------p---kl-- 115
XP_013604348.1    -------------d-..-d-------a---.--------------- 127
XP_013622750.1    ------q------d-t.dnsn---------.-------p---kl-- 115
XP_013712275.1    ------q------d-t.dnsn---------.-------p---kl-- 128
XP_009106163.1    -------------d-..-d-----------.--------------- 127
XP_009106171.1    -------------d-..-d-----------.--------------- 127
XP_009135872.1    ------q--------t.dnsn---------.-------p---kl-- 128
XP_009135873.1    ------q--------t.dnsn---------.-------p---kl-- 128
XP_013597765.1    ---------------t.-d-----------x--------------- 125
XP_002868346.1    ----------s----..id----v---a--.-------t------- 127
XP_002883484.1    -----------g---..-------------.-------t-----vq 131
NP_187053.2       ----y--sknlngqannsf-kigimtmm-ektpdadeiq-enlsig 138
NP_566744.1       -----------g---..-------------.-------t--m---q 131
NP_567408.1       ----------s----..id----v---a--.-------t----l-- 127
NP_974356.1       -----------g---..-------------.-------t--m---q 131
NP_001031626.1    ----------s----..id----v---a--.-------t----l-- 127
NP_001118567.1    ----y--sknlngqannsf-kigimtmm-ektpdadeiq-enlsig 138
NP_001046045.2    --------k-ph--fs.ehsv-e-aamemq.rnaa---g....img 103
NP_001047838.1    ----y---k-nk-dtg.l-a-rg-faahgi..sfa-aapptips-e 121

BjMYB1            QEQNEGYQVTEALRAQMEVQRRLHEQLEYGQVQRRLQLRIEAQGKY 102
XP_013712274.1    -----s----------------------h--------v-------- 174
XP_013730350.1    ----------------------------vqr...------------ 169
XP_013748880.1    ----------------------------h----------------- 173
XP_013748881.1    .............................................. 120
XP_013604275.1    ----------------------------h----------------- 173
XP_013622751.1    -----s----------------------...------v-------- 158
XP_013604348.1    ----------------------------vqr...------------ 170
XP_013622750.1    -----s----------------------h--------v-------- 161
XP_013712275.1    -----s----------------------...------v-------- 171
XP_009106163.1    ----------------------------h----l------------ 173
XP_009106171.1    ----------------------------vq...l------------ 170
XP_009135872.1    -----s----------------------h--------v-------- 174
XP_009135873.1    -----s----------------------...------v-------- 171
XP_013597765.1    ------------------------k---vqr...------------ 168
XP_002868346.1    -----s-----------------------a---------------- 173
XP_002883484.1    ---------------------k------h----------------- 177
NP_187053.2       pqp-knspig---qm-i-----------lr.........------- 175
NP_566744.1       ------------------------d---...--------------- 174
NP_567408.1       -----s----------------------...--------------- 170
NP_974356.1       ------------------------d--------------------- 177
NP_001031626.1    -----s-----------------------t---------------- 173
NP_001118567.1    pqp-knspig---qm-i-----------vqr...h----------- 181
NP_001046045.2    rsm-hdrn-nd-i-m-------------...--kh--m-------- 146
NP_001047838.1    nnnagetplad---y-i----k------...--kk--m-------- 164

BjMYB1            LQSILEKACQ..AFDDQAAAFVGLEAAREELSELAIKVS....... 139
XP_013712274.1    --t------k..---e---m-t---t-------------nns.... 214
XP_013730350.1    ---------k..---------------------------nss.... 209
XP_013748880.1    ---------k..---------------------------nss.... 213
XP_013748881.1    ............---------------------------nss.... 150
XP_013604275.1    ---------k..---------------------------nss.... 213
XP_013622751.1    --t------k..---e---m-t---t-------------nns.... 198
XP_013604348.1    ---------k..---------------------------nss.... 210
XP_013622750.1    --t------k..---e---m-t---t-------------nns.... 201
XP_013712275.1    --t------k..---e---m-t---t-------------nns.... 211
```

```
XP_009106163.1    ---------k..---------------------------nss.... 213
XP_009106171.1    ---------k..---------------------------nss.... 210
XP_009135872.1    ---------k..--ee---m-t---t-------------nss.... 214
XP_009135873.1    ---------k..--ee---m-t---t-------------nss.... 211
XP_013597765.1    ---------k..---e---t-a--------------r--ns..... 207
XP_002868346.1    ---------k..-iee--v--a-----------------isngc.. 215
XP_002883484.1    ---------k..---e-----a----------d------nss.... 217
NP_187053.2       ---v----qe..tlgr-nlgaa-i---kvq----vs---aeypnss 219
NP_566744.1       ---------k..---e---t-a-----------------nss.... 214
NP_567408.1       ---------k..-iee--v--a---------------a-itngc.. 212
NP_974356.1       ---------k..---e---t-a-----------------nss.... 217
NP_001031626.1    ---------k..-iee--v--a---------------a-itngc.. 215
NP_001118567.1    ---v----qe..tlgr-nlgaa-i---kvq----vs---aeypnss 225
NP_001046045.2    m-------y-tl-ag-v---vacgp-gyks-gnhqaa-ldvcsmgf 192
NP_001047838.1    --t-----qn..nlsyd-tgtan---t-tq-tdfnlal-g...... 202

BjMYB1            .........................QGTAVPFLDATK......... 151
XP_013712274.1    .........................---t--yf----m........ 227
XP_013730350.1    .........................-------f-s--......... 221
XP_013748880.1    .........................-------f-t--......... 225
XP_013748881.1    .........................-------f-t--......... 162
XP_013604275.1    .........................-------f-t--......... 225
XP_013622751.1    .........................---t--yf----m........ 211
XP_013604348.1    .........................-------f-t--......... 222
XP_013622750.1    .........................---t--yf----m........ 214
XP_013712275.1    .........................---t--yf----m........ 224
XP_009106163.1    .........................-------f-t--......... 225
XP_009106171.1    .........................-------f-t--......... 222
XP_009135872.1    .........................--at--yf----m........ 227
XP_009135873.1    .........................--at--yf----m........ 224
XP_013597765.1    ...........................-r---f----......... 217
XP_002868346.1    .........................---tnt.f-t--......... 226
XP_002883484.1    .........................---t--yf----......... 229
NP_187053.2       flepkelqnlcsqqmqtnyppdcslescltssegtq-nskmlenn. 264
NP_566744.1       .........................---s--yf----......... 226
NP_567408.1       .........................---tst.f-t--......... 223
NP_974356.1       .........................---s--yf----......... 229
NP_001031626.1    .........................---tst.f-t--......... 226
NP_001118567.1    flepkelqnlcsqqmqtnyppdcslescltssegtq-nskmlenn. 270
NP_001046045.2    pslqdlhmyggaggghldlqqqqppas-mes-facgdgggslgkta 238
NP_001047838.1    ........................fmnnvsqvceqnn......... 215

BjMYB1            ...........MMMMPSLS..................ELEVA.... 164
XP_013712274.1    ...........--------..................-----a... 241
XP_013730350.1    ...........--------..................--a--.... 234
XP_013748880.1    ...........------v-..................--a--.... 238
XP_013748881.1    ...........------v-..................--a--.... 175
XP_013604275.1    ...........------v-..................--a--.... 238
XP_013622751.1    ...........--------..................-----a... 225
XP_013604348.1    ...........------v-..................--a--.... 235
XP_013622750.1    ...........--------..................-----a... 228
XP_013712275.1    ...........--------..................-----a... 238
XP_009106163.1    ...........--------..................--a--.... 238
XP_009106171.1    ...........--------..................--a--.... 235
XP_009135872.1    ...........--------..................-----a... 241
XP_009135873.1    ...........--------..................-----a... 238
XP_013597765.1    ...........--------..................--s--.... 230
```

```
XP_002868346.1     ............-tl----..................--a--.... 238
XP_002883484.1     ...........--------..................--t--.... 242
NP_187053.2        ..........rlglrtyigdstseqkeimeeplfqrm--twteg.. 298
NP_566744.1        ...........--------..................--a--.... 239
NP_567408.1        ............--i----..................--a--.... 235
NP_974356.1        ...........--------..................--a--.... 242
NP_001031626.1     ............--i----..................--a--.... 238
NP_001118567.1     ..........rlglrtyigdstseqkeimeeplfqrm--twteg.. 304
NP_001046045.2     aktrhyggagksp--wgvddddddddpagkcgggghhq-qm-pppm 284
NP_001047838.1     ...........gelakai-..................-dnlr.... 228

BjMYB1             ....IDTKNNITT.NCSVESSLTSNTNGS.....SVSAASMKKRHR 200
XP_013712274.1     ....--h-s----t---------------.....------------ 278
XP_013730350.1     ....v--------.---------------.....----------l- 270
XP_013748880.1     ....v-----t--.---------------.....-l--------l- 274
XP_013748881.1     ....v-----t--.---------------.....-l--------l- 211
XP_013604275.1     ....v--------.---------------.....-l--------l- 274
XP_013622751.1     ....--h-s----t---------------.....------------ 262
XP_013604348.1     ....v--------.---------------.....-l--------l- 271
XP_013622750.1     ....--h-s----t---------------.....------------ 265
XP_013712275.1     ....--h-s----t---------------.....------------ 275
XP_009106163.1     ....v--------.---------------.....----------l- 274
XP_009106171.1     ....v--------.---------------.....----------l- 271
XP_009135872.1     ....--h-s----t---------------.....------------ 278
XP_009135873.1     ....--h-s----t---------------.....------------ 275
XP_013597765.1     ....--h-t----.------p--------.....-i---------- 266
XP_002868346.1     ....-eh--.....---a------s-v--.....p---gl----q- 270
XP_002883484.1     ....--n------.----------i----.....-i--------q- 278
NP_187053.2        ....lrgnpyls-mvsea-qrisyserspgrlsig-glhgh-sq-q 340
NP_566744.1        ....--n------.----------i-h--.....-i--------q- 275
NP_567408.1        ....-eh--.....---a------s-v--.....p----l----q- 267
NP_974356.1        ....--n------.----------i-h--.....-i--------q- 278
NP_001031626.1     ....-eh--.....---a------s-v--.....p----l----q- 270
NP_001118567.1     ....lrgnpyls-mvsea-qrisyserspgrlsig-glhgh-sq-q 346
NP_001046045.2     mdgg--vmdslaadvyetkpimsgdst--kgggyd-a--as-lerp 330
NP_001047838.1     ...ttnlgfqlyhgiqdsddvkc-qde-lllldlnikgggydhlss 271

BjMYB1             G.....DDVGLGYEAGWIVP.SSTIG.................... 220
XP_013712274.1     -......gnnv---gs-t--.-----.................... 297
XP_013730350.1     -.....-----------n--s-----.................... 291
XP_013748880.1     -.....------------m-s-----.................... 295
XP_013748881.1     -.....------------m-s-----.................... 232
XP_013604275.1     -.....------------m-s-----.................... 295
XP_013622751.1     -......gnnv---gs-t--.-----.................... 281
XP_013604348.1     -.....------------m-s-----.................... 292
XP_013622750.1     -......gnnv---gs-t--.-----.................... 284
XP_013712275.1     -......gnnv---gs-t--.-----.................... 294
XP_009106163.1     -.....-----------n--s-----.................... 295
XP_009106171.1     -.....-----------n--s-----.................... 292
XP_009135872.1     -......gdnv---gs-t--.-----.................... 297
XP_009135873.1     -......gdnv---gs-t--.-----.................... 294
XP_013597765.1     .......-m--------pns.-----.................... 283
XP_002868346.1     -vfgng-s-vv-h-v--vm-.--s--.................... 295
XP_002883484.1     -.....-nl-v---s---m-.-----.................... 298
NP_187053.2        qgnnedhkletrnrk-mdstteldlnthvenycttrtkqfdlngfs 386
NP_566744.1        -.....-nl-v---s---m-.-----.................... 295
NP_567408.1        -vfgng-s-vv-hd---vm-.--s--.................... 292
```

```
NP_974356.1        -.....-nl-v---s---m-.-----................... 298
NP_001031626.1     -vfgng-s-vv-hd---vm-.--s--................... 295
NP_001118567.1     qgnnedhkletrnrk-mdstteldlnthvenycttrtkqfdlngfs 392
NP_001046045.2     s...prrppq--spsvmagaqtrnlsyg................. 355
NP_001047838.1     n.......amr-g-s-lkisqhrr..................... 288

BjMYB1             ..                                            220
XP_013712274.1     ..                                            297
XP_013730350.1     ..                                            291
XP_013748880.1     ..                                            295
XP_013748881.1     ..                                            232
XP_013604275.1     ..                                            295
XP_013622751.1     ..                                            281
XP_013604348.1     ..                                            292
XP_013622750.1     ..                                            284
XP_013712275.1     ..                                            294
XP_009106163.1     ..                                            295
XP_009106171.1     ..                                            292
XP_009135872.1     ..                                            297
XP_009135873.1     ..                                            294
XP_013597765.1     ..                                            283
XP_002868346.1     ..                                            295
XP_002883484.1     ..                                            298
NP_187053.2        wn                                            388
NP_566744.1        ..                                            295
NP_567408.1        ..                                            292
NP_974356.1        ..                                            298
NP_001031626.1     ..                                            295
NP_001118567.1     wn                                            394
NP_001046045.2     ..                                            355
NP_001047838.1     ..                                            288
```

Fig. S1 Alignments of amino acid sequences of BjMYB1 and similar MYB-type proteins from *Brassica*, *Arabidopsis* and *Oryza*. The software Clustalx 1.83 was used to make the identity comparison. The amino acid sequence of BjMYB1 was used as the reference sequence in capital letters. '-' dash lines indicate amino acid identical to those of BjMYB1. '.' indicate absence of amino acid. The letters in lowercase show the amino acids different from those of BjMYB1. Numbers on the right indicate the positions of amino acids in each protein. The percentage numbers in shadow show identity percentages between *Bj*MYB1 and the orthologs.

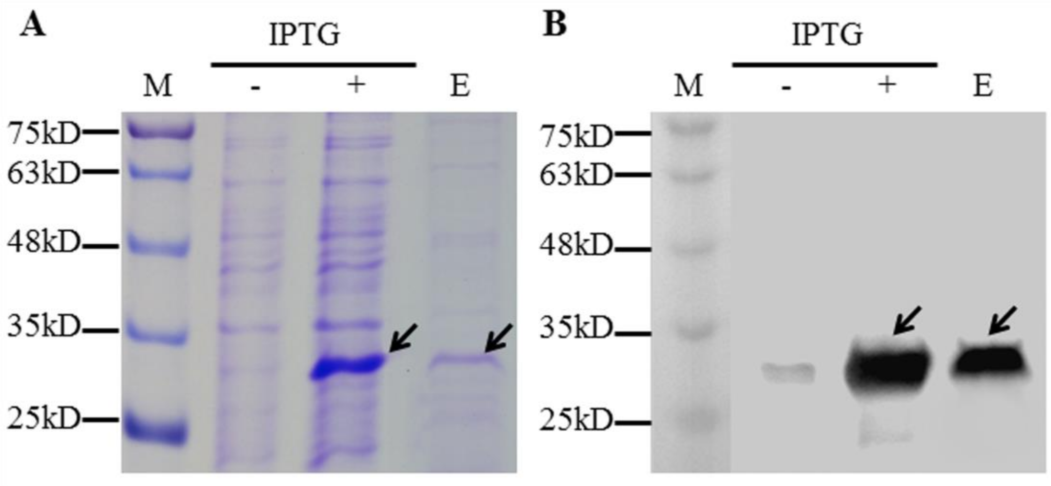

Fig. S2. Expression of His-BjMYB1 fusion protein in *E. coli* Rosetta (DE3). (A) Coomassie blue staining of SDS-PAGE gel. "M" indicates the protein marker. "E" indicates the eluate in purifying the His-BjMYB1 fusion protein. "+" indicates induction with 0.6mM IPTG. "-" indicates no IPTG induction. The arrows indicate the band of His-BjMYB1. (B) Western blot analysis of His-BjMYB1 with His as antibody. The arrows indicate the blotting band of His-BjMYB1.

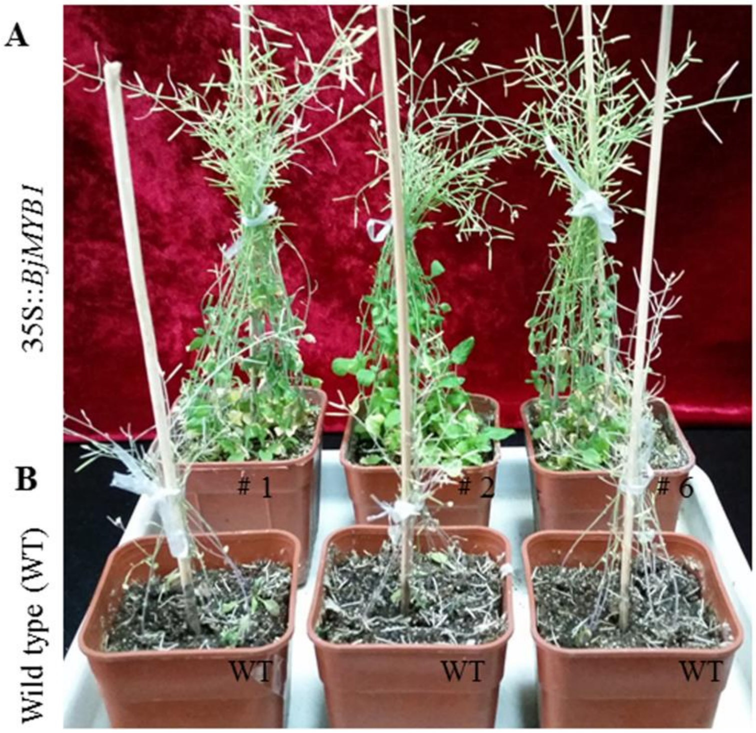

Fig. S3. *BjMYB1*-overexpressing *Arabidopsis* plants exhibited enhanced disease resistance to *B. cinerea* at mature stage. (A) Phenotypes of the three *BjMYB1*-overexpressing *A. thaliana* lines (#1, #2 and #6) at mature stage post inoculation with *B. cinerea*. (B) Phenotypes of the wild-type Col-0 (WT) at mature stage post inoculation with *B. cinerea*.

A

W1: 5´ - agtccaaaataa**caaaTGACaatt**ccacctttattttaac-3´ / Wbl-1 element
W2: 5´ - agggtggtacag**tcagTGACtcct**gagcttcagagagggt-3´ / Wbl-2 element
W3: 5´ - gtagtccagcag**ctagTGACtctg**ctagagatagtgtggt-3´ / Wbl-3 element
W5: 5´ - agggtggtccct**ctagTGACtcat**gagctagagagagggt-3´ / Wbl-5 element
W6: 5´ - caaatgaacttg**gtggTGACaaaa**tagaagtttagaatgt-3´ / Wbl-6 element

B

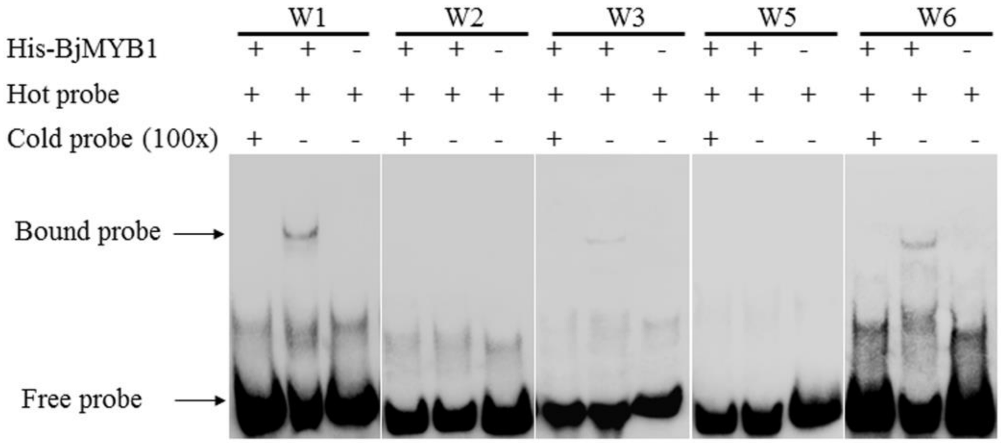

Fig. S4. EMSA analyses for the interaction between BjMYB1 and other five W box-like (Wbl) elements in BjC-P. (A) Nucleotide sequences of the probes with Wbl elements shown in bold and underlined. W1, W2, W3, W5 and W6 are BjC-P fragments containing the Wbl-1, Wbl-2, Wbl-3, Wbl-5 and Wbl-6 element, respectively. (B) Electrophoretic mobility shift assay (EMSA) for the DNA-binding activity of His-BjMYB1 fusion protein with W1, W2, W3, W5 and W6 fragments (probes). An equal amount of BjMYB1 protein or hot probe (biotin-labeled) was used in all lanes. Cold probes (without biotic-labeled) is 100 times of the hot probes as binding competitors. The bound and free hot probes are indicated by arrows on the left.
